# *MiR-17* Partly Promotes Hematopoietic Cell Expansion through Augmenting HIF-1α in Osteoblasts

**DOI:** 10.1371/journal.pone.0070232

**Published:** 2013-07-25

**Authors:** Yuxia Yang, Wei Ma, Dan Wu, Yu Huang, Hongge Li, Junhua Zou, Yanju Zhang, Meifu Feng, Jianyuan Luo

**Affiliations:** 1 Department of Medical Genetics, School of Basic Medical Sciences, Peking University, Beijing, China; 2 Institute of Zoology, Chinese Academy of Sciences, Beijing, China; 3 Department of Histology and Embryology, Capital University of Medical Sciences, Beijing, China; 4 Department of Medical and Molecular Genetics, School of Medicine, Indiana University, Indianapolis, Indiana, United States of America; 5 Tianjin Central Hospital for Obstetrics and Gynecology, Tianjin, China; 6 Department of Medical & Research Technology, Department of Pathology, School of Medicine, University of Maryland, Baltimore, Maryland, United States of America; French Blood Institute, France

## Abstract

**Background:**

Hematopoietic stem cell (HSC) regulation is highly dependent on interactions with the marrow microenvironment, of which osteogenic cells play a crucial role. While evidence is accumulating for an important role of intrinsic *miR-17* in regulating HSCs and HPCs, whether *miR-17* signaling pathways are also necessary in the cell-extrinsic control of hematopoiesis hereto remains poorly understood.

**Methodology/Principal Findings:**

Using the immortalized clone with the characteristics of osteoblasts, FBMOB-hTERT, *in vitro* expansion, long-term culture initiating cell (LTC-IC) and non-obese diabetic/severe combined immunodeficient disease (NOD/SCID) mice repopulating cell (SRC) assay revealed that the ectopic expression of *miR-17* partly promoted the ability of FBMOB-hTERT to support human cord blood (CB) CD34^+^ cell expansion and maintain their multipotency. It also seemed that osteoblastic *miR-17* was prone to cause a specific expansion of the erythroid lineage. Conversely, deficient expression of *miR-17* partly inhibited the hematopoietic supporting ability of FBMOB-hTERT. We further identified that *HIF-1α* is responsible for, at least in part, the promoted hematopoietic supporting ability of FBMOB-hTERT caused by *miR-17*. *HIF-1α* expression is markedly enhanced in *miR-17* overexpressed FBMOB-hTERT upon interaction with CB CD34^+^ cells compared to other niche associated factors. More interestingly, the specific erythroid lineage expansion of CB CD34^+^ cells caused by osteoblastic *miR-17* was abrogated by *HIF-1α* knock down.

**Conclusion/Significance:**

Our data demonstrated that CB CD34^+^ cell expansion can be partly promoted by osteoblastic *miR-17,* and in particular, ectopic *miR-17* can cause a specific expansion of the erythroid lineage through augmenting *HIF-1α* in osteoblasts.

## Introduction

Hematopoietic stem cells (HSCs) are multipotent progenitor cells that give rise to all types of mature blood cells. Tracer studies of transplanted HSCs reveal that they most likely reside in bone cavities specifically adjacent to endosteal bone lined by osteoblast cells [1], [2], [3]. HSCs share an important relationship with osteoblasts and other stromal elements of the bone marrow niche critical to their maintenance and protection [1], [4], [5]. Furthermore, it is now widely accepted that gradients of oxygen from below 1% in hypoxic niches to 6% in the sinusoidal cavity exist within the human bone marrow, which also keeps HSCs in a low proliferative and relatively quiescent state [6], [7], [8]. Proliferating progenitors are distributed in O_2_-rich areas [9], [10], [11], [12]. In line with these reports, Rankin *et al* have recently showed that the HIF signaling pathway from osteoblasts play key roles in hematopoiesis [13]. Collectively, this evidence suggests that the interaction between HSCs and osteoblasts, forming specialized hypoxia, is crucial in keeping the HSC pool size *in vivo* and to prevent exhaustion of HSCs from uncontrolled cell-cycle entry and excessive proliferation.

MicroRNAs (miRNAs) are short non-coding RNAs comprised of 21 to 23 nucleotides in length that post-transcriptionally regulate mRNA expression [14]. Involvement of miRNAs in hematopoiesis is strongly suggested by the position of miRNA genes near translocation breakpoints and by their presence in loci targeted for deletion in human leukemias [15]. Moreover, expression profiling data suggest a major role for miRNAs in the regulation of hematopoietic cell commitment, proliferation, apoptosis, survival and differentiation [16], [17], [18]. Most of the studies that have been performed so far on miRNA expression in hematopoietic stem and progenitor cells focus on hematopoietic lineage differentiation [19], [20], [21]. *MiR-17* (also called *miR*-*17*-*5p*), an important member of the *miR-17-92* cluster [22], is expressed abundantly in hematopoietic progenitors and promotes hematopoietic cell expansion by targeting sequestosome 1 (sqstm1) regulated pathways in mice [23]. Consistent with this data, expression of *miR-17* is detected in human CD34^+^ cells and is shown to be significantly down-regulated during *in vitro* differentiation toward mature megakaryocytes, monocytes and monocytopoiesis [17], [24]. Collectively, these examples illustrate a more general role for the autocrine production of *miR-17* as a regulator of critical pathways determining normal hematopoietic cell fate and differentiation. While evidence is accumulating for a crucial role of intrinsic *miR-17* in regulating HSCs and HPCs, whether *miR-17* signaling pathways within the hematopoietic niche, especially in osteoblasts, are also necessary in the cell-extrinsic control of hematopoiesis has not yet been examined. Interestingly, one group recently found that some miRNAs are expressed differently between two stromal cell lines that have distinguishable functional characteristics and gene expression profiles for hematopoiesis, suggesting a potential role for miRNAs in regulating hematopoietic cell migration and niche function [25]. Related to this, two other separate studies described a regulatory role for miRNAs in controlling the expression of hematopoietic niche associated genes in endothelial cells [26], [27].

We have previously reported one immortalized clone with the characteristics of osteoblasts [28], designated as FBMOB-hTERT, derived from human fetal bone marrow stromal cells with retroviral vectors containing the human telomerase catalytic subunit (hTERT) gene [28]. The FBMOB-hTERT cells support the human cord blood (CB) HSCs and HPCs expansion and maintain their self-renewal and multipotency [28]. Using these cells, we found that *miR-17* was significantly overexpressed. The ectopic expression of *miR-17* partly promoted the ability of FBMOB-hTERT to support human CB CD34^+^ cell expansion and maintain their self-renewal and multipotency. It is noted that ectopic *miR-17* in FBMOB-hTERT preferentially supports a specific expansion of the erythroid lineage. Conversely, *miR-17* knockdown in FBMOB-hTERT suppressed the hematopoietic supporting ability of FBMOB-hTERT, in particular the mature erythroid cell growth. We further identified that *HIF-1α* is responsible for, at least in part, the promoted function of ectopic *miR-17* in FBMOB-hTERT on hematopoiesis. The expression of *HIF-1α* was significantly enhanced in *miR-17* overexpressed FBMOB-hTERT upon interaction with CB CD34^+^ cells compared with other niche associated factors such as *KL*, *SDF-1* and *EPO*. More interestingly, selective expansion of the erythroid lineage of CB CD34^+^ cells through osteoblastic *miR-17* was abrogated by *HIF-1α* knock down, demonstrating that *HIF-1α* was, at least partly, a mediator of *miR-17*-induced CB CD34^+^ cell expansion in FBMOB-hTERT.

In summary, in addition to intracellular *miR-17*, our data raised the possibility that *miR-17* was also necessary in the cell-extrinsic control of HSCs and HPCs function, which is, at least in part, through the augmented *HIF-1α* signal pathways.

## Materials and Methods

### Cell Cultures

The hTERT-transduced fetal bone marrow osteoblasts (FBMOB-hTERT) [28] and cryopreserved primary bone marrow stromal cells (BMSCs) [28] were thawed and plated in DMEM (Gibco) containing 10% FBS (Gibco) at 37°C supplemented with 5% CO_2,_ as described previously [28]. The Phoenix cell line, obtained from American Type Culture Collection (ATCC) was also cultured in the same medium. Human CB samples were obtained as described previously [28]. Briefly, CB mononuclear cells (MNCs) were isolated by the lymphocyte separation medium (1.077 g/ml) (TBD Biotech, Tianjing, China), and were immunomagnetically enriched for CD34^+^ cells using the MACS CD34^+^ Cell Isolation Kit (Miltenyi Biotech Inc., Bergisch Gladbach, Germany) according to the manufacturer’s instructions. The purity of CD34^+^ cells was about 80%–90%, as determined by flow cytometry (FCM). This study was approved by the Ethics Committee of Peking University. Before the experiments, the subjects were informed of the objectives, requirements and procedures of the experiments. All subjects gave informed written consent to participate in the study.

### Construction and Packaging of shRNA Vectors

The two *miR-17*-specific small hairpin RNAs (17/KD and 17/KD1) and *HIF-1α*-specific shRNA (HIF1α/KD) oligomers [29] were tested. The details of the shRNA sequences are provided in **Table 1**. Sense and antisense oligomers were used to produce double-stranded oligomers, and the oligomers were inserted into the retroviral vector RNAi-pSIREN-RetroQ, which drives shRNA production from the U6 promoter and also contains puromycin resistance (Clontech). Inserts were confirmed by sequencing. If not otherwise mentioned, RNAi-pSIREN-RetroQ vectors containing scrambled target sequences not complementary to any known miRNA were served as controls for 17/KD or HIF1α/KD (CTRL). The Phoenix packaging cell line was co-transfected with the RNAi-pSIREN-RetroQ retroviral plasmid and the viral packaging plasmid by Lipofectanine 2000 (Invitrogen) following the manufacturer’s instructions. Viral supernatants were collected at 48 or 72 hours after transfection and stored at −80°C for future use.

**Table 1:** Primers used in this study.

Name	Sequence
**shRNA-I**	5′-GGATCC**GTCAAAGTGCTTACAGTGCAGG**TTCAAGAGACCTGCACTGTAAGCACTTTGACTTTTTTGAATTC-3′
**shRNA-II**	5′-GGATCC**GTGCATCTACTGCAGTGAAGGC**TTCAAGAGAGCCTTCACTGCAGTAGATGCACTTTTTTGAATTC-3′
**shRNA-III**	5′-GGATCC**GCTGGAGACACAATCATATCTT**TTCAAGAGAAAGATATGATTGTGTCTCCAGCTTTTTTGAATTC-3′
**Scrambled shRNA**	5′ -GGATCCACACGTCCGAACATACTACTTCAAGAGAGTAGTATGTTCGGACGTGTTTTTTTGAATTC-3′
**pre-miR-17**	5′ -CGGATCCAGTTTGAGGTGTTAAT-3′; 5′ -GGAATTCACTATCTGCACTAGATG-3′
**Hsa-miR17**	5′ -AGTGCGTGTCGTGGAGTC-3′; 5′ -GCAAAGTGCTTACAGTGCA-3′
**U6**	5′ -GCTTCGGCAGCACATATACTAAAAT-3′; 5′ -CGCTTCACGAATTTGCGTGTCAT-3′
**HIF-1α**	5′ -CCATTAGAAAGCAGTTCCGC-3′; 5′ - TGGGTAGGAGATGGAGATGC -3′
**SDF-1**	5′- AACGCCAAGGTCGTGGTCGTGCTG-3′; 5′ - CACATCTTGAACCTCTTGTTTAAAAGC -3′
**KL**	5′- GACAGCTTGACTGATCTTCTGGAC-3′; 5′ - ACTGCTGTCATTCCTAAGGGAGCT -3′
**EPO**	5′- GAGAATATCACGACGGGCTG -3′; 5′ - CCACTGACGGCTTTATCCAC -3′
**17α-satellite**	5′- ACGGGATAACTGCACCTAAC -3′; 5′ - CCATAGGAGGGTTCAACTCT -3′

### Cloning of Human *pre-miR-17* Gene, the Derivative Construction and Retroviral Infection

Total DNA was isolated using DNA Mini kit (Watson Biotechnologies, Inc., Shanghai, China) according to the manufacturer’s instructions. Primers for amplifying human *pre-miR-17* gene were provided in **Table 1**. PCR was carried out following standard protocols. The full-length human *pre-miR-17* expression clone was inserted into the retroviral vector, yielding the expression constructs, pCMV-*pre-miR-17* (17/OE). If not otherwise mentioned, vectors containing the empty intron sequence served as controls for 17/OE (CTRL). Viral supernatants were produced and stored as described as above.

The retroviral supernatant (1.0 ml) containing indicated retrovirus and polybrene (8ug/ml) was added to culture dishes when indicated cells grew up to 70%–80% confluent. After incubation for 5 hours at 37°C, the transduction medium was replaced by 2.0 ml DMEM, and puromycin (4ug/ml) was added to the cultures 24 hours later. Puromycin resistant clones grew out after 4–5 weeks of culture, and the pool cells were transferred to a fresh dish for expansion.

### RNA Extraction and Real-time RT-PCR

Total RNA was extracted using Trizol reagent (Invitrogen). Real-time RT-PCR analysis was performed with a Bio-Rad iCycler using the iQSYBER green supermix (Bio-Rad). U6 snRNA (for miRNA) or GAPDH (glyceraldehyde-3-phosphate dehydrogenase) (for mRNA) was used as the endogenous control. Standard curves for internal control and tested genes were measured each time to determine the relative level of the respective transcript. The copy number was normalized to endogenous control levels.

### Antibodies and Western Blotting

Cells were washed twice with phosphate buffered saline (PBS) and resuspended in RIPA buffer with protease inhibitor, and allowed to lyse on ice for 30 min. After lysis, samples were cleared by centrifuging at 15,000×*g* for 15 min at 4°C and supernatants were collected. Protein concentrations of supernatants were determined using a BCA (bicinchoninic acid) Protein Assay kit (Pierce). Samples were analyzed by western blotting using standard procedures. Mouse monoclonal GAPDH (Santa Cruz) was used at 1:5000. Rabbit anti-HIF-1α (Santa Cruz) was used at 1:1000. The blots were visualized by an enhanced chemiluminescence (ECL) kit following the manufacturer’s instructions. To ensure equal protein loading, each membrane was probed with the GAPDH antibody.

### Luciferase Assay

17/OE FBMOB-hTERT or CTRL cells were seeded in 24-well plates at a density of 1.0×10^5^ cells per well and were allowed to grow for 24 hours before transfection. Afterward, each well was transiently transfected with 900 ng pGL3 of either wild-type or mutant 3′UTR of *HIF-1α* [30] with a pRLTK vector (100 ng) for normalization of transfection efficiency using lipofectamine 2000. Cell lysates were harvested 36 hours post transfection, and firefly and renilla luciferase activities were measured by the Dual-Luciferase Reporter Assay System (Promega). The value of relative luciferase activity denotes the firefly luciferase activity normalized to that of renilla for each assay. Three independent experiments were done in triplicate.

### Co-culture of Stromal Cells with Human CB CD34^+^ Cells

Co-culture assay was performed as described previously [28]. Briefly, the indicated stromal cells were irradiated at a dose of 40 Gy, and seeded in 24-well plates (5.0×10^5^/well) overnight. CD34^+^ cells (2.0×10^4^/well) in Iscove’s Modified Dulbecco’s Medium (IMDM) (Bio-WHITTAKER) were supplemented with 10% FBS, 10^−4^M 2-mercaptoethanol, 2 mM L-glutamine, 5 mg/ml insulin, 100 U/ml penicillin, and 100 mg/ml streptomycin as well as a cytokine cocktail consisting of Flt ligand (FL; 10 ng/ml), SCF (10 ng/ml), thrombopoietin (TPO, 10 ng/ml), and interleukin (IL)-6 (10 ng/ml), all of which were purchased from Peprotech Inc. After 14 days of culture, non-adherent and adherent hematopoietic cells that were loosely attached to stromal cells were harvested by gentle pipetting, counted, and analyzed for CD34 and CD38 expression by flow cytometry.

### Long-term Culture Initiating Cells (LTC-IC) Assay

LTC-IC was analyzed as described previously [28]. Briefly, 1.0×10^4^ CB CD34^+^ cells were plated into six-well plates containing a nearly confluent, irradiated indicated stromal cell monolayer in LTC medium (Myelo-Cult, StemCell Inc., Vancouver, BC, Canada) along with 10^−6^M hydrocortisone sodium hemisuccinate (Sigma). The LTC medium contained horse serum, fetal bovine serum, 2-Mercaptoethanol and α-MEM. 50% of the medium was replaced weekly with fresh medium. Both non-adherent and adherent cells were harvested weekly during 5–8 weeks of culture, and were cultured in the complete methylcellulose medium containing 2.8% BSA, 30% FBS (Hyclone), 50 ng/ml SCF, 20 ng/ml IL-3, 20 ng/ml GM-CSF, 20 ng/ml IL-6, and 3 U/ml EPO (Kirin, Tokyo, Japan), at 37°C with 5% CO_2_. After 14–16 days of cultures, colonies with greater than 50 cells were counted to assess LTC-IC activities. Colony-forming units (CFU-Cs), colony-forming unit-mix (CFU-Mix’s), colony-forming unit-erythrocyte (CFU-Es) and burst-forming unit-erythrocyte (BFU-Es) were counted to calculate the frequency of LTC-IC according to the manufacturer’s instructions (StemCell Inc.).

### NOD/SCID Repopulating Cell (SRC) Assay

5.0×10^4^ CB CD34^+^ cells were co-cultured with indicated stromal cells for 4 weeks, harvested as described above, and injected intravenously (i.v.) into 8-week-old, sublethally irradiated (3.5 Gy) NOD/SCID mice. Positive control mice were transplanted with freshly isolated CD34^+^ cells. The mice were sacrificed 12 weeks post-transplantation. Bone marrow mononuclear cells were harvested and analyzed by flow cytometry. Human cell repopulation was assessed using anti-human-CD45-FITC (fluorescein isothiocyanate), anti-human-CD34-PE (phycoerythrin), anti-human-CD36-PC7 (PE-cyanin 7) and anti-glycophorin A (GPA)-APC (allophycocyanin) antibodies. Isotype controls were mouse IgG1 conjugated to PE, APC PC7 or FITC. All antibodies were from Becton Dickinson. PCR amplification of the human 17α-satellite gene was employed as a second test for the presence of human cells in the NOD/SCID mice that had received transplants. In cases where secondary transplants were performed, bone marrow cells were prepared from primary recipients as described below, pelleted 4 minutes at 720 g and resuspended at 1.6 to 2.4 ×10^6^/mL in IMDM/10% FBS. Secondary recipients each received 6.5 ×10^5^ human CD34^+^ cells from primary recipients. All care and handling of animals were performed with the approval of Institutional Authority for Laboratory Animal Care of Peking University.

### Statistical Analysis

The results are expressed as the mean ± standard deviation (SD). Statistical comparisons were performed using a two-tailed Student’s *t* -test.

## Results

### *MiR-17* is Endogenously Expressed in FBMOB-hTERT and Primary BMSCs

**Figure 1:**
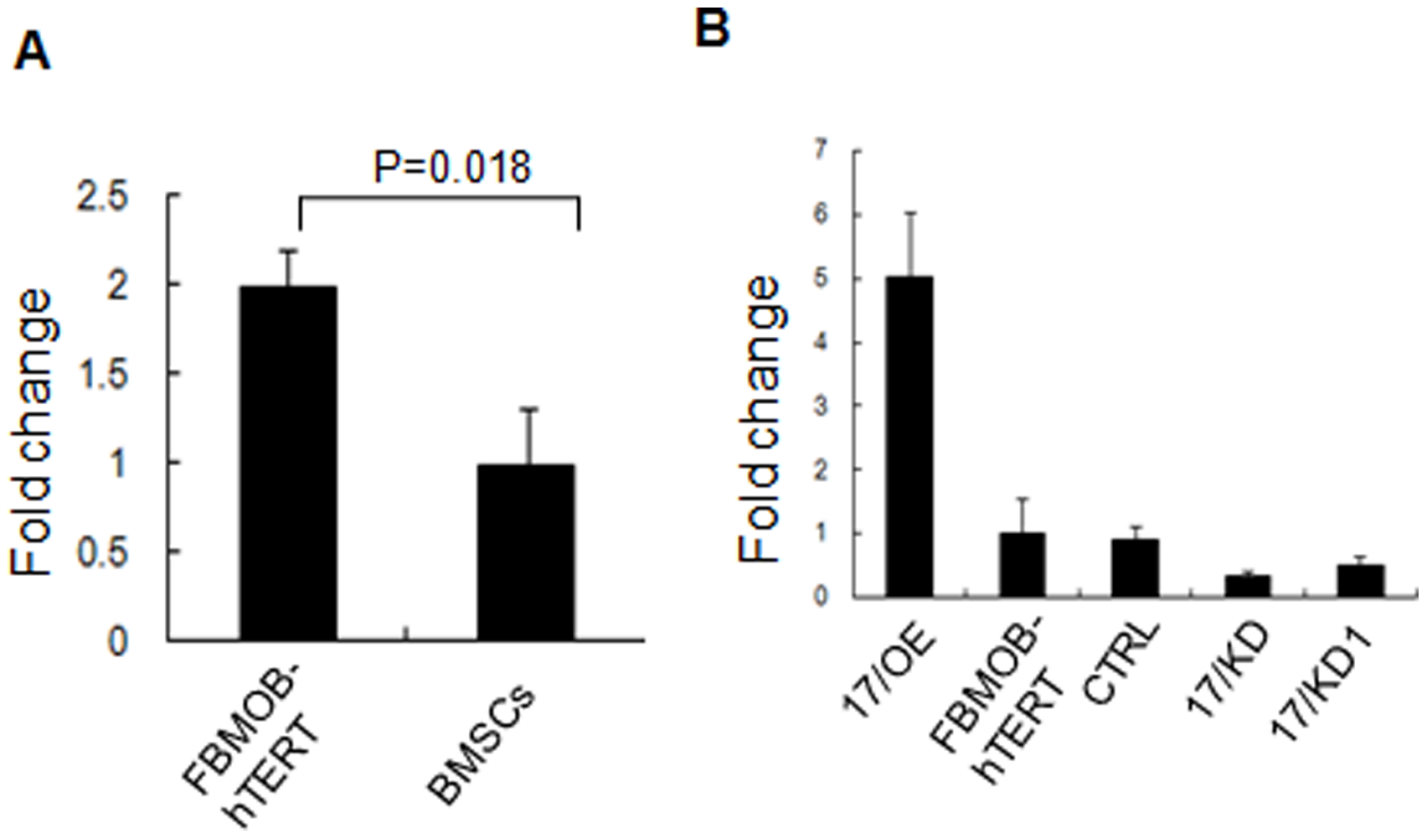
The expression of *miR-17* in FBMOB-hTERT cells. **A:** The expression level of *miR-17* in FBMOB-hTERT cells was evaluated by real-time RT-PCR. Each reaction was performed in triplicate. The data are presented as the ratio of *miR-17* levels (relative to U6) in FBMOB-hTERT to that in bone marrow stromal cells (BMSCs). **B:** Real-time RT-PCR was performed to evaluate the expression level of *miR-17* in FBMOB-hTERT cells after retrovirally transduced with vectors for *miR-17* overexpression (17/OE), *miR-17* knockdown (17/KD or 17/KD1), or control (CTRL). The data are presented as the ratio of *miR-17* levels (relative to U6) in 17/OE, FBMOB-hTERT, 17/KD or 17/KD1 to that in CTRL. Each reaction was performed in triplicate.

To test our hypothesis that osteoblastic *miR-17 *may influence hematopoiesis, we first determined the *miR-17* expression level in the FBMOB-hTERT and primary bone marrow stromal cells (BMSCs). Real-time RT-PCR assay showed that *miR-17* is endogenously expressed in these cells. Compared with primary BMSCs, FBMOB-hTERT expressed a significantly higher level of *miR-17* (**Figure 1A**). *MiR-17* is also endogenously expressed in cord blood (CB) CD34^+^ cells (data not shown), which is consistent with the previous reports [16], [23]. The higher expression of *miR-17* in FBMOB-hTERT cells was of interest given that such an expression was likely to have hematopoietic functional consequences.

### The Function of osteoblastic *miR-17 *on Expansion of CB CD34^+^ Cells

To analyze the function of osteoblastic *miR-17* on the expansion of HSCs and HPCs, the *miR-17* overexpressing and knockdown models were created using FBMOB-hTERT cells by using retroviral vectors. The *miR-17* levels in the FBMOB-hTERT cells transduced with the indicated virus were determined using real-time RT-PCR. We observed obvious up and down-modulation of *miR-17* in 17/OE cells and 17/KD cells respectively compared to the CTRL (**Figure 1B**). Based on shRNA influence on *miR-17* expression, 17/KD was chosen for further studies.

We first investigated the ability of the osteoblastic *miR-17* to expand CB CD34^+^ cells *in vitro*. CD34^+^-enriched CB cells were co-cultured with irradiated 17/OE, 17/KD, or CTRL cells. After 14 days of culturing, the numbers of total cells, CD34^+^ cells, CD34^+^CD38^−^ cells, and CD34^+^CD38^+^ cells were counted, respectively. As shown in **Table 2**, although both CD34^+^CD38^−^ and CD34^+^CD38^+^ cells were expanded dramatically in the presence of FBMOB-hTERT, the 17/OE cells appeared to be more potent than CTRL cells (CD34^+^CD38^−^ cells: 24.21- versus 18.45-folds) in supporting CD34^+^CD38^−^ cell expansion. Conversely, deficient expression of *miR-17 *in FBMOB-hTERT suppressed CD34^+^CD38^−^ cell expansion (CD34^+^CD38^−^ cells: 13.27- versus 18.45-folds). These results suggest that *miR-17* in FBMOB-hTERT cells can promote CD34^+^CD38^−^ cell expansion *in vitro*.

**Table 2:** *Ex vivo* expansion of CB CD34^+^ cells over 14 days.

	17/OE	CTRL	17/KD
Total cells	80.01±10.11	82.13±8.18	86.29±5.02
CD34^+^ cells	30.14±4.23*	24.65±3.89	17.01±5.41*
CD34^+^CD38^−^ cells	24.21±3.05*	18.45±2.31	13.27±3.11*
CD34^+^CD38^+^ cells	92.67±9.01	92.16±12.87	91.88±7.90

The cells were stained with PE-conjugated mAb to CD34 and FITC-conjugated mAb to CD38, and analyzed by flow cytometry. Values indicate the fold increase compared with the initial number of cells (2.0×10^4^/well).The results are given as mean ± standard deviation (SD) (*n* = 6). ^*^p<0. 05 versus the CTRL cells (Student’s *t*-test).

**Figure 2:**
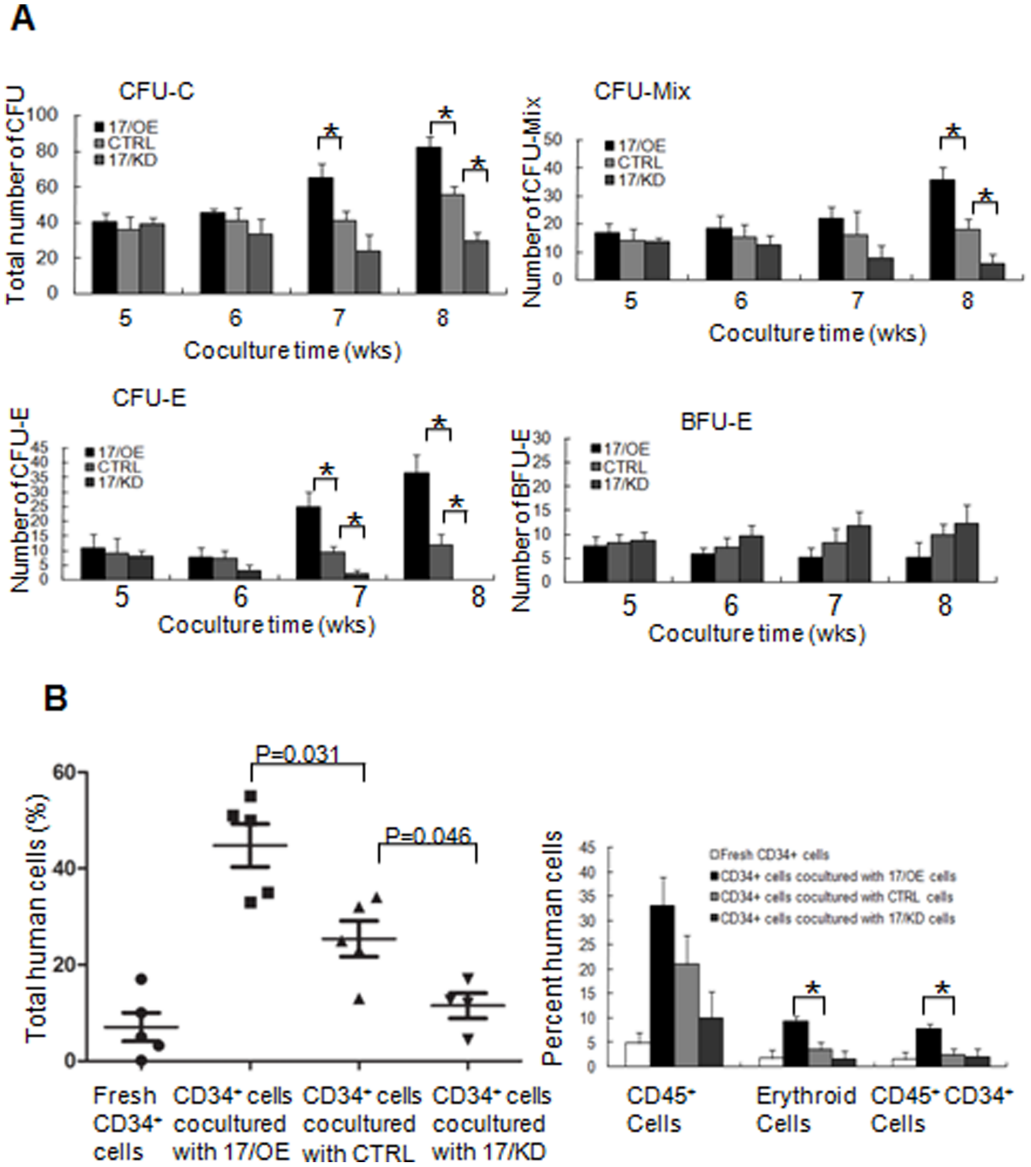
The effect of *miR-17* modulation in FBMOB-hTERT cells on CB CD34^+^ cells. **A:** The effect of *miR-17* modulation in FBMOB-hTERT cells on long-term culture initiating cells activity of CB CD34^+^ cells. 1.0×10^4^ CD34^+^ CB cells were co-cultured with FBMOB-hTERT cells after transduced with vectors for *miR-17* overexpression (17/OE), *miR-17* knockdown (17/KD), or control (CTRL) for 5-8 weeks and then subject to colony-forming-unit (CFU) assay. After 14-16 days of culture, the colonies, including CFU-Mixs, CFU-E, and BFU-E with greater than 50 cells were counted. The results are expressed as mean ± SD (n = 6). *p<0.05, compared between 17/OE or 17/KD, and CTRL group (Student’s *t*-test). **B:** Effect of *miR-17* modulation in FBMOB-hTERT cells on repopulation of CB CD34^+^ cells in non-obese diabetic/severe combined immunodeficient disease (NOD/SCID) mice. 5.0×10^4 ^CB CD34^+^ cells were co-cultured with 17/OE, 17/KD or CTRL, harvested at 4 weeks of culture and then injected intravenously into the sublethally irradiated NOD/SCID mice (n = 6 per group). The mice were sacrificed 12 weeks after transplantation and the mononuclear cells from bone marrow were analyzed for human cells composed of CD45^+^, CD45^–^CD36^+^ and CD36^–^GPA^+^ cells and CD45^+^CD34^+^ population by flow cytometry. The level of total human cell engraftment was shown in the left panel. P = 0.031 or 0.046, compared between the NOD/SCID mice injected with CD34^+^ cells co-cultured with 17/OE or 17/KD and those injected with CD34^+^ cells co-cultured with CTRL (Student’s *t*-test). The fraction of CD45^+^, erythroid (CD45^−^CD36^+^ and CD36^−^GPA^+^) and CD45^+^CD34^+^ population among the engrafted human cells was shown in right panel. *p<0.05, compared between the NOD/SCID mice injected with CD34^+^ cells co-cultured with 17/OE and those injected with CD34^+^ cells co-cultured with CTRL (Student’s *t*-test). The significant difference was only analyzed between the mice injected with CD34^+^ cells co-cultured with 17/OE or 17/KD and those injected with CD34^+^ cells co-cultured with CTRL.

To test the capability of osteoblastic *miR-17* in supporting self-renewal and maintaining multipotent differentiation of HSC, we performed a Long-term culture initiating cells (LTC-IC) assay. CB CD34^+^cells were co-cultured with 17/OE, 17/KD, or CTRL cells in LTC-IC medium for 5–8 weeks, and then subject to a CFU assay. After 14–16 days of culturing, the colonies with more than 50 cells were counted. As shown in **Figure 2A**, the number of total CFCs from the cells co-cultured with 17/OE cells for 7 or 8 weeks was significantly higher than that of the cells co-cultured with the CTRL. Moreover, after co-cultured for 8 weeks, the number of total CFCs from the cells co-cultured with 17/KD cells became significantly lower compared to the cells co-cultured with the CTRL, suggesting that osteoblastic *miR-17* partly supports the LTC-IC activity of HSC. Interestingly, the number of mature erythroid (CFU-E) from the cells co-cultured with 17/OE cells for 7 or 8 weeks was significantly higher than that of the cells co-cultured with the CTRL, whereas only after co-cultured for 8 weeks, the number of total CFU-Mix’s from the cells co-cultured with 17/OE cells was significantly higher than that of the cells co-cultured with the CTRL, suggesting that ectopic *miR-17* in FBMOB-hTERT preferentially supports a specific expansion of the erythroid lineage. Knockdown of *miR-17 *in FBMOB-hTERT cells, on the other hand, resulted in reduced hematopoietic support, which was followed by diminishing CFU output. It is noted that compared to a significant and specific increase or decrease in the number of CFU-E from cells co-cultured with 17/OE or 17/KD cells for 7 or 8 weeks than that from cells co-cultured with the CTRL, the number of immature erythroid (BFU-E) progenitors did not change significantly after osteoblastic *miR-17* modulation.

**Figure 3:**
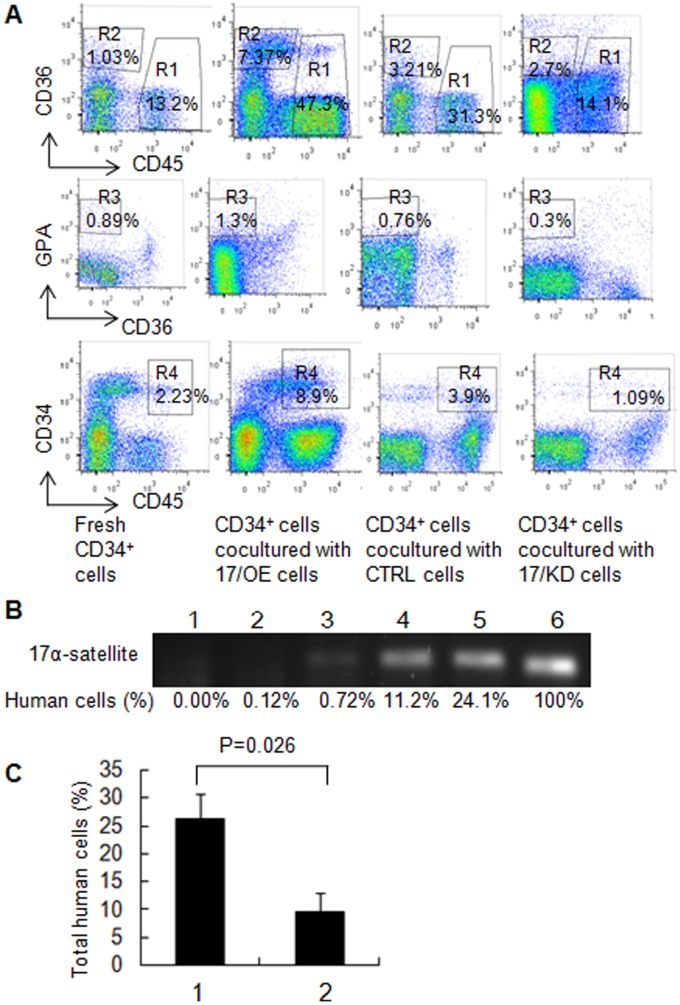
Effect of *miR-17* modulation on repopulation of CD34^+^ cells in primary and secondary NOD/SCID mice. A: Flow cytometry analysis of the human CB CD34^+^ cell repopulation in a representative primary NOD/SCID mouse after co-cultured with 17/OE, 17/KD or CTRL cells. Fresh CD34^+^ cells were served as controls. The mononuclear cells from bone marrow harvested from the engrafted NOD/SCID mice were examined by flow cytometry for the assessment of human cells composed of CD45^+^ cells (R1) and erythroid cells including CD45^–^CD36^+^ (R2) and CD36^–^GPA^+^ (R3) population and CD45^+^CD34^+^ cells (R4). **B:** The bone marrow mononuclear cells (MNCs) containing the different percentage of human cells (lanes 2-5) from the primary representative engrafted mice were analyzed for human-specific 17α-satellite DNA by PCR. The human-specific 17α-satellite gene was detected when the human cells were over 0.70% (lanes 3–6) whereas it was indetectable at a percentage of 0.12% (lane 2). Lane 1, one mouse without transplants; lane 2, one mouse receiving transplants of fresh CD34^+^ cells; lane 3, one mouse receiving transplants of CD34^+^ cells co-cultured with 17/KD cells; lane 4, one mouse receiving transplants of CD34^+^ cells co-cultured with CTRL cells; lane 5, one mouse receiving transplants of CD34^+^ cells co-cultured with 17/OE cells; lane 6 indicates positive control (human peripheral blood (PB) MNCs). **C:** Effect of *miR-17* modulation in FBMOB-hTERT cells on repopulation of CB CD34^+^ cells in secondary NOD/SCID mice. Human cells were analyzed in secondary mice 12 weeks after intravenous injection of bone marrow cells from primary mice, which were injected with CB CD34^+^ cells co-cultured with 17/OE or CTRL for 4 weeks and sacrificed 8 weeks after transplantation. P = 0.026, compared between 1 and 2 (1, bone marrow mononuclear cells in secondary mice from primary mice injected with CB CD34^+^ cells co-cultured with 17/OE; 2, bone marrow mononuclear cells in secondary mice from primary mice injected with CB CD34^+^ cells co-cultured with CTRL) (Student’s *t*-test).

To further support our *in vitro* expansion and LTC-IC results, we examined the engraftment of CB CD34^+^ cells after co-cultured with 17/OE, 17/KD, or CTRL cells in NOD/SCID mice. The sublethally irradiated NOD/SCID mice were transplanted with 5.0×10^4^ CB CD34^+^ cells from the co-cultures with irradiated 17/OE, CTRL, or 17/KD cells for 4 weeks. The level of total human cell engraftment composed of CD45^+^, CD45^–^CD36^+^ and CD36^–^GPA^+^ cells, and CD45^+^CD34^+^ populations were assessed in the bone marrow mononuclear cells of the graft mice at 12 weeks post-transplant by flow cytometry. At 12 weeks, the degree of total human cells from the mice injected with CD34^+^ cells co-cultured with 17/OE or 17/KD was significantly higher or lower than that of the mice injected with CD34^+^ cells co-cultured with CTRL (**Figure 2B left panel**; p = 0.031 and 0.046 respectively). The statistical analysis was only done in comparison between the mice injected with CD34^+^ cells co-cultured with 17/OE or 17/KD and those injected with CD34^+^ cells co-cultured with CTRL. We further analyzed the multilineage development from input CD34^+^ populations. Flow cytometry analysis of the human graft in a representative graft mouse from each group is shown in **Figure 3A**. There was no significant difference in the percentage of CD45^+^ cells by comparing the 17/OE or 17/KD group with the corresponding CTRL group (**Figure 2B right panel**). However, we observed a significantly higher percentage of erythroid cells, including CD45^–^CD36^+^ and CD36^–^GPA^+^ populations in 17/OE group compared to the percentage in the CTRL group. Similarly, compared to the CTRL group, the 17/OE group also showed a significantly higher percentage of CD45^+^CD34^+^ cells (**Figure 2B right panel**). The observed multilineage development from input CD34^+^ populations co-cultured with 17/OE cells coincides with expansion and LTC-IC assays *in vitro*. Whereas the percentage of CD45^+^CD34^+^ cells and erythroid cells in the 17/KD group whose transplants of CD34^+^ cells had been co-cultured with 17/KD cells showed a tendency, although this was not significant, to be lower than that of mice receiving transplants of CD34^+^ cells co-cultured with CTRL cells. To further confirm that the human cells determined by flow cytomtry were of human origin, the human-specific 17α-satellite gene was detected by PCR in several representative graft mice, which contained different percentages of human cells. We found that the human 17α-satellite gene could be detected by PCR amplification when the percentage of human cells was over 0.72% (**Figure 3B**, lanes 3–6), whereas it was indetectable at a percentage of 0.12% (**Figure 3B**, lanes 2). These results partly confirmed our *in vitro* data and suggest that osteoblastic *miR-17* partly promotes the ability of FBMOB-hTERT to maintain the multipotency of CB CD34^+^ cells *in vitro.* It also seemed that osteoblastic *miR-17* is prone to cause a specific expansion of the erythroid lineage.

To further confirm whether or not the human cell fraction in the primary engraftment contained NOD/SCID repopulating cells (SRCs), we collected bone marrow from the primary mice that received a transplant of CD34^+^ cells co-cultured with 17/OE cells or CTRL cells and transplanted the marrow into sublethally irradiated secondary recipients. In these experiments, the bone marrows (BMs) of six mice from the two groups were transplanted into eight recipients (calculated human CD34^+^ cells received/mouse was 6.5×10^5^). Human cells in the BM from the secondary recipients consisting of CD45^+^, CD45^–^CD36^+^ and CD36^–^GPA^+^ populations and the CD45^+^CD34^+^ cells were also analyzed by flow cytometry. While the cells from the two groups could engraft secondary recipients, the percentage of total human cell engrafting the BM from the primary recipient transplanted with CD34^+^ cells co-cultured with 17/OE, as demonstrated by flow cytometry, was significantly higher than that of the primary recipient transplanted with CD34^+^ cells co-cultured with the CTRL (**Figure 3C**). Although only a limited number of secondary recipients have been analyzed, these data suggest that the ability of FBMOB-hTERT to maintain the multipotency of CB CD34^+^ cells *in vitro* was partly promoted through *miR-17* over-expression.

### *MiR-17* Up-regulates *HIF-1α* Expression upon Interaction with CB CD34^+^ Cells

**Figure 4:**
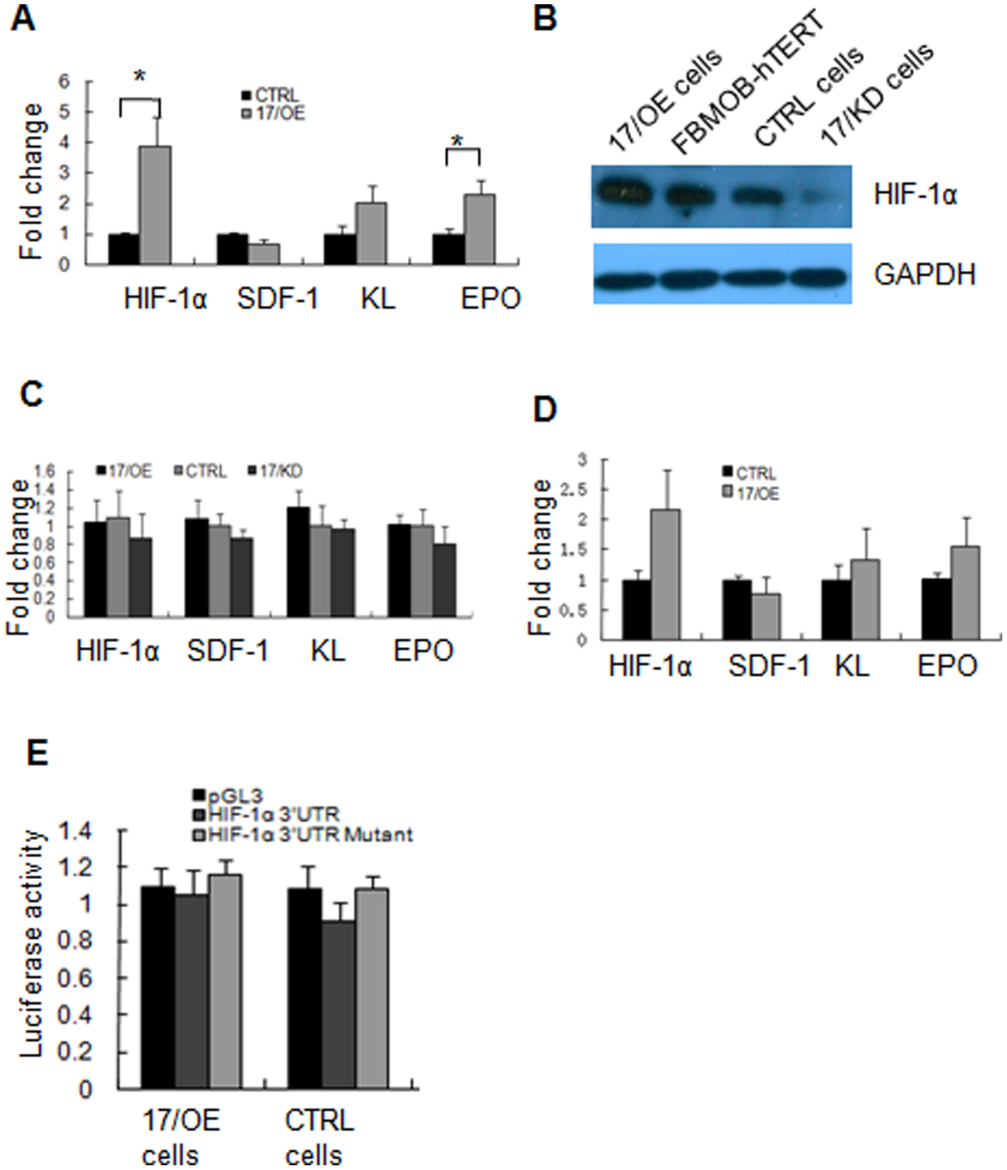
*MiR-17 *up-regulates *HIF-1α* expression upon interaction with CB CD34^+^ cells. **A:** 1.0×10^6^ 17/OE or CTRL cells were co-cultured with CB CD34^+^ cells (1.0×10^4^/well) in six-well plates for 24 hours, and then washed twice to remove loosely adherent and non-adherent cells. The adherent cells were harvested and analyzed by real-time RT-PCR for transcripts of the niche associated genes: HIF-1α, SDF-1, KL and EPO. The results are expressed as mean ± SD (n = 3). *p<0.05, compared between 17/OE cells and CTRL cells (Student’s *t*-test). **B:** HIF-1α protein levels were measured by western blotting in 17/OE, 17/KD or CTRL cells after interaction with CB CD34^+^ cells for 24 hours. **C:** The transcripts of the niche associated genes: *HIF-1α*, *SDF-1*, *KL* and *EPO* were analyzed by real-time RT-PCR in 17/OE, 17/KD or CTRL cells. The results are expressed as mean ± SD (n = 3). Without CB CD34^+^ cell existing, the expression of the indicated niche associated genes was not changed significantly in 17/OE or 17/KD cells comparing with that in CTRL cells. **D.** The transcripts of the niche associated genes were analyzed by real-time RT-PCR in bone marrow stromal cells (BMSCs) with over-expressed *miR-17* and the control cells upon interaction with CB CD34^+^ cells. The expression of *HIF-1α* was up-regulated after *miR-17 *over-expressed whereas the expression of *SDF-1*, *KL* and *EPO* did not changed obviously. The results are expressed as mean ± SD (n = 3). **E:** Luciferase reporter assays to check whether *miR-17* directly target *HIF-1α* in FBMOB-hTERT. The luciferase activities were not significantly decreased in 17/OE cells compared with that in CTRL cells. The results are given as mean ± standard deviation (SD) (*n* = 3).

To explore the mechanisms by which *miR-17* promotes the function of FBMOB-hTERT in supporting hematopoiesis thus causing a specific expansion of the erythroid lineage, we examined the production of hematopoietic supporting growth factors including the hypoxia-inducible transcription factor (*HIF-1α*), stromal cell-derived factor (*SDF-1*), stem cellfactor/c-kit ligand (*SCF/KL*) and erythropoietin (*EPO*) by 17/OE and CTRL cells during interaction with CB CD34^+^ cells. The 17/OE and CTRL cells were co-cultured with or without 1.0×10^4^ CD34^+^ cells for 24 hours. The adherent co-cultured cells were harvested after washing off the loosely adherent and non-adherent cells, and analyzed by real-time RT-PCR or western-blotting. Among these genes, *HIF-1α* and *EPO* were found to be significantly increased in 17/OE cells after interaction with CB CD34^+^ cells compared to that in CTRL cells (**Figure 4A** **,** about 4 and 2 folds respectively). In contrast, *KL* exhibited no significant increase and there was almost no change in *SDF-1* expression in 17/OE cells after interaction with CB CD34^+^ cells compared to that in CTRL cells. Protein expression of HIF-1α was also measured by western-blotting. As shown in **Figure 4B**, the levels of HIF-1α protein were significantly up-regulated in 17/OE cells after co-cultured with CB CD34^+^ cells compared to that in CTRLs. We also confirmed the up-regulation of *HIF-1α* in primary BMSCs after ectopic expression of *miR-17 *upon interaction with CB CD34^+^ cells. These results suggest that ectopic *miR-17* in FBMOB-hTERT augmented the expression of niche associated genes during co-cultured with CB CD34^+^ cells, which may be responsible for the hematopoietic supporting ability of osteoblastic *miR-17*.

### *HIF-1α* Knock Down Partially Abrogate the Hematopoietic Supporting Ability of Osteoblastic *miR-17*

**Figure 5:**
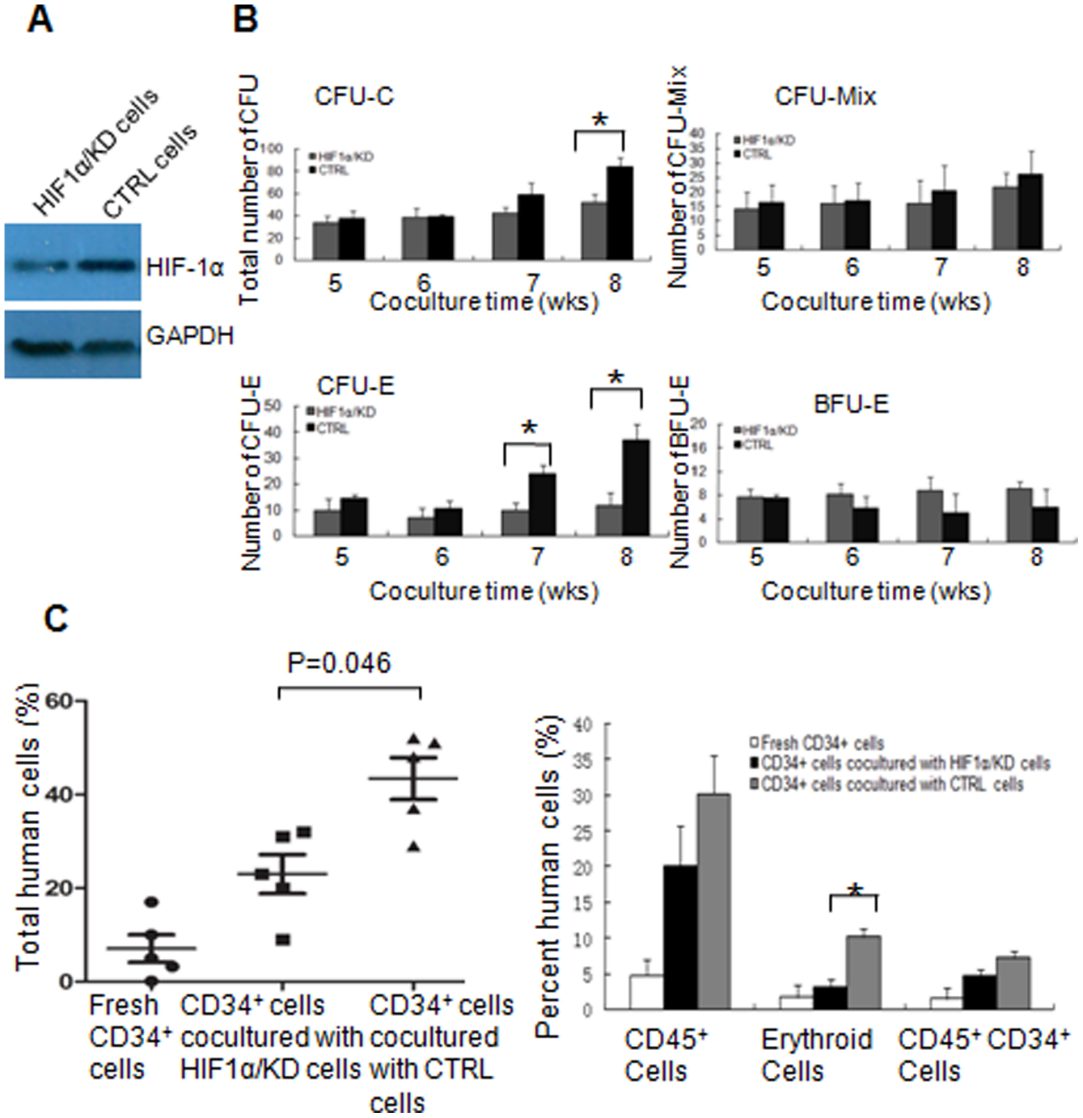
HIF-1α knockdown partially abrogates the hematopoietic supporting ability of osteoblastic *miR-17*. **A:** Western blotting was performed to evaluate the expression level of HIF-1α protein in 17/OE cells after transduced with vectors for HIF1α knockdown (HIF1α/KD), or control (CTRL). **B:** The effect of the HIF1α/KD cells on long-term culture initiating cells activity of CB CD34^+^ cells. 1.0×10^4^ CD34^+^ CB cells were co-cultured with HIF1α/KD cells or CTRL for 5-8 weeks and then subject to CFU assay. After 14–16 days of culture, the colonies, including CFU-Mixs, CFU-E, and BFU-E with greater than 50 cells were counted. The results are expressed as mean ± SD (n = 6). *p<0.05, compared between HIF1α/KD and CTRL group (Student’s *t*-test). **C:** Effect of HIF1α/KD cells on repopulation of CB CD34^+^ cells in non-obese diabetic/severe combined immunodeficient disease (NOD/SCID) mice. 5.0×10^4 ^CB CD34^+^ cells were co-cultured with HIF1α/KD or CTRL cells, harvested at 4 weeks of culture and then injected intravenously into the sublethally irradiated NOD/SCID mice (n = 6 per group). The mice were sacrificed 12 weeks after transplantation and the mononuclear cells from bone marrow were analyzed for human cells composed of CD45^+^, CD45^–^CD36^+^ and CD36^–^GPA^+^ cells and CD45^+^CD34^+^ population by flow cytometry. The level of total human cell engraftment was shown in the left panel. p = 0.046, compared between the mice injected with CD34^+^ cells co-cultured with HIF1α/KD and those injected with CD34^+^ cells co-cultured with CTRL (Student’s *t*-test). The fraction of CD45^+^, erythroid (CD45^−^CD36^+^ and CD45^−^ CD36^−^GPA^+^) and CD45^+^CD34^+^ cells among the engrafted human cells was shown in the right panel. *p<0.05, compared between the mice injected with CD34^+^ cells co-cultured with HIF1α/KD and those injected with CD34^+^ cells co-cultured with CTRL (Student’s *t*-test). The significant difference was only analyzed between the mice injected with CD34^+^ cells co-cultured with HIF1α/KD and those injected with CD34^+^ cells co-cultured with CTRL.

Since ectopic *miR-17* in FBMOB-hTERT cells can significantly up-regulate *HIF-1α* upon interaction with CB CD34^+^ cells, we examined whether or not the hematopoietic supporting ability of osteoblastic *miR-17* is dependent on the augmented *HIF-1α* activity in FBMOB-hTERT cells. The *HIF-1α* knockdown model was created using 17/OE FBMOB-hTERT cells by stably expressing *HIF-1α* shRNA or nonspecific shRNA (termed HIF1α/KD and CTRL respectively) via retroviral transduction. We performed western-blotting to detect HIF-1α protein levels in the retrotransduced 17/OE cells. As shown in **Figure 5A**, the level of HIF-1α protein was significantly knocked down by *HIF-1α* shRNA in HIF1α/KD cells when compared to levels in CTRL cells.

Using the cells above, we further investigated the hematopoietic supporting ability of osteoblastic *miR-17* after *HIF-1α* knock down. The *in vitro* expansion assay (**Table 3**) demonstrated that CTRL cells appeared to be more potent than HIF1α/KD cells (CD34^+^CD38^−^ cells: 24.83- versus 15.65-folds) in supporting CD34^+^CD38^−^ cell expansion. The LTC-IC (**Figure 5B**) assay showed that, only after co-cultured for 8 weeks, the total number of CFCs from the cells co-cultured with CTRL cells was significantly higher than that of the cells co-cultured with HIF1α/KD cells. There was no significant difference between the number of CFU-Mix’s from the cells co-cultured with CTRL cells, and from the cells co-cultured with HIF1α/KD cells. These suggested that the hematopoietic supporting ability of osteoblastic *miR-17* was partially abrogated by *HIF-1α* knock down. It is interesting that the number of mature erythroid (CFU-Es) from the cells co-cultured with the CTRL for 7 or 8 weeks was significantly higher than that of the cells co-cultured with HIF1α/KD, which further suggested that the specific erythroid lineage expansion of CB CD34^+^ cells caused by osteoblastic *miR-17* was abrogated by *HIF-1α* knock down. The number of immature erythroid (BFU-E) progenitors from the cells co-cultured with HIF1α/KD cells did not change significantly.

**Table 3:** *Ex vivo* expansion of CB CD34^+^ cells over 14 days.

	HIF1α/KD	CTRL
Total cells	82.09±7.16	80.27±9.03
CD34^+^ cells	21.91±4.52*	30.02±3.99
CD34^+^CD38^−^ cells	15.65±4.12*	24.83±4.03
CD34^+^CD38^+^ cells	92.33±11.30	91.98±8.31

The effect of *ex vivo* expansion of HIF1α/KD cells on CB CD34^+^ cells was assayed according to the methods described above.

* p<0. 05 versus CTRL cells (*n* = 6) (Student’s *t*-test).

**Figure 6:**
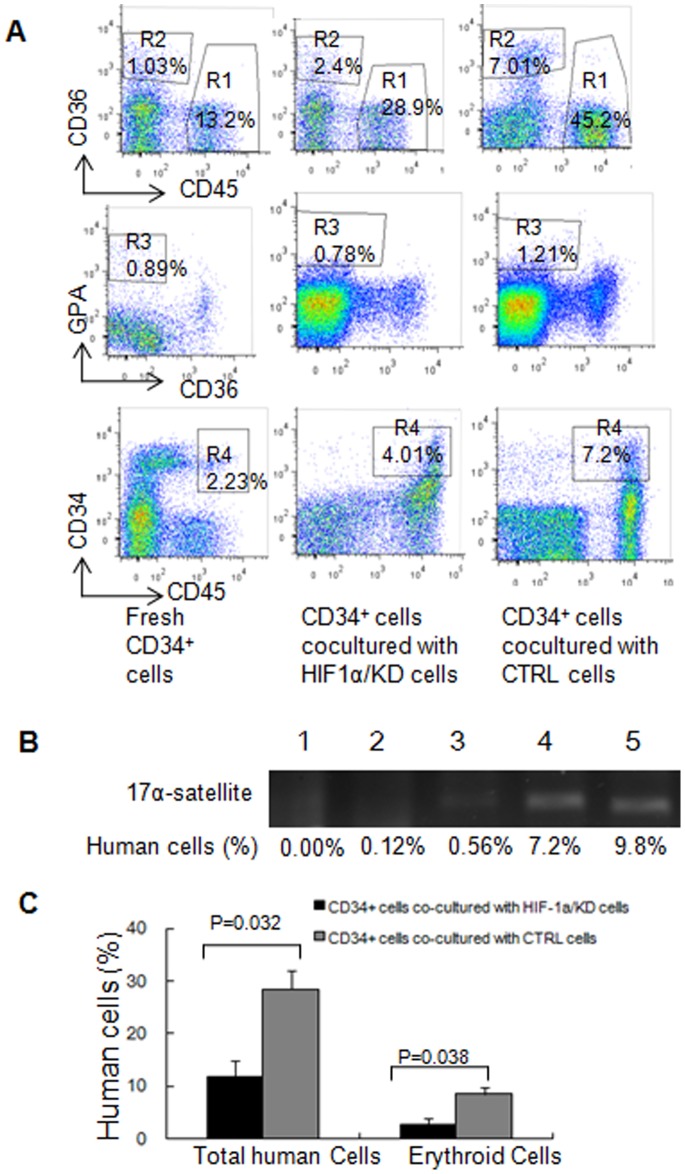
Repopulation of CD34^+^ cells after co-cultured with HIF1α/KD in primary and secondary NOD/SCID mice. **A:** Flow cytometry analysis of the human CD34^+^ cell repopulation in a representative primary NOD/SCID mouse after co-cultured with HIF1α/KD or CTRL. Fresh CD34^+^ cells were served as controls. The mononuclear cells from bone marrow harvested from the primary injected NOD/SCID mice were examined by flow cytometry for the assessment of human cells composed of CD45^+^ cells (R1) and erythroid cells including CD45^–^CD36^+^ (R2) and CD36^–^GPA^+^ population (R3) and CD45^+^CD34^+^ cells (R4). **B:** The bone marrow mononuclear cells (MNCs) containing the different percentage of human cells (lanes 2-5) from the primary representative engrafted mice were analyzed for human-specific 17α-satellite DNA by PCR. The human-specific 17α-satellite gene was detected when the human cells were over 0.50% (lanes 3-5), whereas it was indetectable at a percentage of 0.12% (lanes 2). Lane 1, one mouse without transplants; lanes 2-3, two mice receiving transplants of fresh CD34^+^ cells; lane 4, one mouse receiving transplants of CD34^+^ cells co-cultured with HIF1α/KD cells; lane 5, one mouse receiving transplants of CD34^+^ cells co-cultured with CTRL cells. **C:** Effect of HIF-1α knockdown in 17/OE cells on repopulation of CB CD34^+^ cells in secondary NOD/SCID mice. Human cells were analyzed in secondary mice 12 weeks after intravenous injection of bone marrow cells from primary mice, which were injected with CB CD34^+^ cells co-cultured with HIF1α/KD or CTRL for 4 weeks and sacrificed 8 weeks after transplantation. The percentage of total human cell or erythroid cells in the secondary BM from the primary recipient transplanted with CD34^+^ cells co-cultured with HIF1α/KD was significant lower than that from the primary recipient transplanted with CD34^+^ cells co-cultured with CTRL (p = 0.032 and 0.038 respectively) (Student’s *t*-test).

To support the above *in vitro* expansion and LTC-IC results, we further examined the engraftment of CB CD34^+^ cells after co-cultured with HIF1α/KD or CTRL cells in NOD/SCID mice (**Figure 5C**). The sublethally irradiated NOD/SCID mice were transplanted with 5.0×10^4^ CB CD34^+^ cells from the co-cultures with irradiated HIF1α/KD or CTRL for 4 weeks. The level of total human cell engraftment composed of CD45^+^, CD45^–^CD36^+^ and CD36^–^GPA^+^ cells, and CD45^+^CD34^+^ population were assessed in the bone marrow mononuclear cells of the engrafted mice at 12 weeks post-transplant by flow cytometry. Flow cytometry analysis of the human graft in a representative graft mouse from each group is shown in **Figure 6A**. At 12 weeks, the degree of total human cells from the mice injected with CD34^+^ cells co-cultured with HIF1α/KD is significantly lower than that in the mice injected with CD34^+^ cells co-cultured with the CTRL (**Figure 5C left panel**; p = 0.046). The significant difference was only analyzed between the mice injected with CD34^+^ cells co-cultured with HIF1α/KD and those injected with CD34^+^ cells co-cultured with CTRL. We further analyzed the multilineage development from input CD34^+^ populations. There was no significant difference in the percentage of CD45^+^ cells (**Figure 5C right panel**) between the two groups; however, we observed a significantly lower percentage of erythroid cells including CD45^–^CD36^+^ and CD36^–^GPA^+^ population in mice injected with CD34^+^ cells co-cultured with HIF1α/KD compared to that in mice injected with CD34^+^ cells co-cultured with the CTRL (**Figure 5C right panel** **)**, which is consistent with the results from the LTC-IC assay *in vitro*. The level of CD45^+^CD34^+^ cells showed no significant difference between the two groups. Human-specific 17α-satellite gene was also detected by PCR in three representative graft mice (**Figure 6B**) to confirm that the human cells determined by flow cytomtry were of human origin. We found that the human 17α-satellite gene could be detected by PCR amplification when the percentage of human cells was more than 0.56% (**Figure 6B**, lanes 3–5), whereas it was indetectable at a percentage of 0.12% (**Figure 6B**, lanes 2). These results confirmed our *in vitro* data and demonstrated that the specific erythroid lineage expansion of CB CD34^+^ cells caused by osteoblastic *miR-17* was abrogated by *HIF-1α* knock down. All these suggested that *HIF-1α* is, at least partly, a mediator of CB CD34^+^ cell expansion caused by *miR-17* in FBMOB-hTERT cells.

To further determine whether the human cell fraction in the primary engraftment contained NOD/SCID repopulating cells (SRCs), we collected bone marrow from the primary mice that received a transplant of CD34^+^ cells co-cultured with HIF1α/KD or CTRL cells and transplanted the marrow into sublethally irradiated secondary recipients. The BMs of six mice from two groups were transplanted into eight recipients (calculated human CD34^+^ cells received/mouse was 6.5×10^5^). Human cells in the BM from the secondary recipients consisting of CD45^+^, CD45^–^CD36^+^ and CD36^–^GPA^+^ populations and the CD45^+^CD34^+^ cells were also analyzed by flow cytometry. The percentage of total human cells or erythroid cells in the secondary BM from the primary recipient transplanted with CD34^+^ cells co-cultured with HIF1α/KD was significantly lower than that of the primary recipient transplanted with CD34^+^ cells co-cultured with the CTRL, as demonstrated by flow cytometry (**Figure 6C**). Although only a limited number of secondary recipients have been analyzed, these data suggest that the specific erythroid lineage expansion of CB CD34^+^ cells caused by osteoblastic *miR-17* was abrogated by *HIF-1α* knock down.

## Discussion

Osteogenic cells, lining in endosteal bone, play a crucial role in regulating HSC function [3], [4]. The FBMOB-hTERT cell line without tumorigenicity has the characteristics of osteoblasts [28] and can actively maintain the capacity of self-renewal and multipotency of HSCs and HPCs [28]. In this study, using FBMOB-hTERT cells [28], we identified that *miR-17 *in FBMOB-hTERT was significantly higher than that in bone marrow stromal cells (BMSCs) (**Figure 1A**), which is possibly responsible for the hematopoietic-supporting characteristic of FBMOB-hTERT. As expected, the expansion and LTC-IC assay *ex vivo* suggested that *miR-17* in FBMOB-hTERT partly promoted the ability of FBMOB-hTERT to support human CB CD34^+^ cell expansion and maintain their multipotency (**Figure 2A**). It seems that the ability of *miR-17* in FBMOB-hTERT to promote CB CD34^+^ cell expansion requires a significant amount of time. This idea is evidenced in the fact that the number of CFU-Mix from the cells co-cultured with 17/OE or 17/KD was significantly higher or lower than that of the cells co-cultured with CTRL cells only after co-cultured for 8 weeks. Although after co-cultured for 5, 6 or 7 weeks, there was a trend toward a decrease or increase in the number of CFU-Mix from the cells co-cultured with 17/KD or 17/OE compared to that from the cells co-cultured with CTRL cells, statistical analyses of the cohort indicated that it did not meet statistical significance (p>0.05). It is of interest to note that osteoblastic *miR-17* seemed to be more prone to support erythroid lineage expansion because the number of mature erythroid (CFU-E) from the cells co-cultured with *miR-17* modulated FBMOB-hTERT for 7 weeks was significantly changed in comparison to the cells co-cultured with CTRL cells. However, compared to a significant and specific change in the number of mature erythroid (CFU-E) from cells co-cultured with 17/OE or 17/KD cells for 7 or 8 weeks to that of cells co-cultured with CTRL, the number of immature erythroid (BFU-E) progenitors from CB CD34^+^ cell did not change significantly after osteoblastic *miR-17* modulation. The function of *miR-17* on CB CD34^+^ cells was confirmed by the primary and secondary engraftment assay in NOD/SCID mice. A significantly higher percentage of human CD45^+^CD34^+^ cells and erythroid cells (CD45^–^CD36^+^ and CD36^–^GPA^+^ cells) was observed in the bone marrow of mice transplanted with CD34^+^ cells co-cultured with 17/OE cells compared to that in the bone marrow of mice transplanted with CD34^+^ cells co-cultured with CTRL cells. In contrast, the percentage of human CD45^+^ cells in the bone marrow of mice whose transplants of CD34^+^ cells had been co-cultured with 17/OE cells showed a tendency, although this was not significant (p>0.05), to be higher than that of mice receiving transplants of CD34^+^ cells co-cultured with CTRL cells. All these suggested that the ectopic *miR-17* signal pathway in FBMOB-hTERT cells may create a niche which can partly promote HSC and HPC expansion and is more suitable for erythroid progenitor differentiation, which subsequently leads to more mature erythroid cells.

The mechanisms underlying the enhanced expansion are largely unclear. One of the mechanisms is likely mediated by a variety of HSC-supporting growth factors, such as *HIF-1α*, which are constitutively activated by overexpressed *miR-17 *upon interaction with CB CD34^+^ cells. In support of this notion, we found that the transcription of *HIF-1α* was significantly up-regulated in 17/OE cells after co-cultured with CB CD34^+^ cells (**Figure 4A**). The protein level of HIF-1α was also up-regulated upon interaction with CB CD34^+^ cells (**Figure 4B**). It seemed that this special environment is vital for the up-regulation of *HIF-1a* caused by *miR-17,* because *HIF-1α* was not changed without the existence of CB CD34^+^ cells regardless of the level of *miR-17* expression (**Figure 4C**). These data suggested that the different expressions of *HIF-1a* in different culture environments were caused by *miR-17*. The microenvironment plays a critical role in the regulation of cell fate and subsequent tissue formation [31], [32], [33] and various signaling molecules were subsequently ignited. Taguchi *et al* [30] suggested that HIF-1α was repressed by miR-17–92 only under a normoxic condition, whereas HIF-1α was robustly induced under hypoxia regardless of the level of miR-17–92 expression [30]. In addition, Jin *et al* [34] found that *miR-17* modulated the diverse effect of canonical Wnt signaling in different microenvironments [34]. On the basis of our results and previous reports, we put forth the idea that different microenvironments lead to different effects of *miR-17* and that mechanism is the key point of our further research.

Except for the environment, the intricate and finely tuned relationship between *HIF-1α* and *miR-17* is also likely dependent on cellular context and appears to be promoter-independent in FBMOB-hTERT. After transfecting pGL3 with the 3′UTR of HIF-1α, the luciferase activities were not significantly decreased in 17/OE cells compared to the activities in CTRL cells (**Figure 4E**). The contradiction with the previous report may be due to the different cellular contexts of non-tumor and tumor cells. Additionally, the mRNA level of *EPO* was also significantly up-regulated in 17/OE, although they were not as high as *HIF-1α*, which was also dependent upon the interaction with CB CD34^+^ cells. *EPO* expression is tightly regulated by developmental, physiological, and cell-type-specific factors during development [35], [36], which can be activated in response to acute anemia or hypoxia to functionally regulate erythropoiesis [13], [37]. Increased *EPO* by *miR-17* may be a feasible explanation for the inclination of 17/OE cells to support the growth of mature erythroid cells. Although the transcript of KL was not significantly altered, further experiments are still needed to explore whether the up-regulated KL, a hematopoietic supporting growth factor [38], is responsible for the promoted hematopoietic supporting ability of 17/OE cells. However, the mRNA levels of *SDF-1*, which is expressed by the niche cellular components and is important for the migration of HSCs [39], was not altered in 17/OE cells with or without interaction with CB CD34^+^ cells. Overall, by changing the expression of hematopoietic supporting factors, ectopic expression of *miR-17* in osteoblastic cells may create a suitable niche for HSC expansion, in particular the specific expansion of the erythroid lineage.

Of great interest, our experiments on *ex vivo* expansion, LTC-IC and SRC *in vivo* assay revealed that selective expansion of the erythroid lineage of CB CD34^+^ cells through osteoblastic *miR-17* was abrogated by *HIF-1α* knock down (**Figure 5**). These data suggested that the function of osteoblastic *miR-17* on HSCs and HPCs was through, at least in part, the *HIF-1α* signaling pathway. Although the relationship between *miR-17* and *HIF-1α* is dependent on the environment and cellular context, our data showed a functional link between *HIF-1α* and *miR-17*, which has also been demonstrated by other research groups [30], [40].

In summary, our data suggested the potential contribution of *miR-17* in bone marrow stem cell niches and an osteoblastic-*miR-17*-*HIF-1α*-HSC crosstalk in hematopoietic development. Our study demonstrated that, in addition to regulating the cellular constituents of HSCs and HPCs, *miR-17* may also participate in the regulation of hematopoietic microenvironment and be involved in intercellular communications between HSCs and their niche cells. Defining the role of *miR-17* in osteoblasts on hematopoiesis raises the possibility that *miR-17* may play a key part in regulating the hematopoietic niche. Further characterization of *miR-17* and other miRNAs on this field will be particularly important, not only for a better understanding of the detailed mechanisms behind HSC self-renewal and lineage commitment, but also for developing novel and efficient molecular targets to prevent and treat hematopoietic disorders.
